# Maize yield prediction and condition monitoring at the sub-county scale in Kenya: synthesis of remote sensing information and crop modeling

**DOI:** 10.1038/s41598-024-62623-w

**Published:** 2024-06-20

**Authors:** Harison K. Kipkulei, Sonoko D. Bellingrath-Kimura, Marcos Lana, Gohar Ghazaryan, Roland Baatz, Custodio Matavel, Mark K. Boitt, Charles B. Chisanga, Brian Rotich, Rodrigo M. Moreira, Stefan Sieber

**Affiliations:** 1 Leibniz Centre for Agricultural Landscape Research (ZALF), Eberswalder Straße 84, 15374 Müncheberg, Germany; 2 Humboldt Universität zu Berlin, Faculty of Life Sciences, Invalidenstraße 42, 10115 Berlin, Germany; 3 Department of Geomatic Engineering and Geospatial Information Systems, Jomo Kenyatta University of Agriculture and Technology, P.O. Box, 62000, Nairobi, 00200 Kenya; 4 Department of Crop Production Ecology, Swedish University of Agricultural Sciences, Box 7043, 75007 Uppsala, Sweden; 5 Geography Department, Humboldt-Universität zu Berlin, Unter Den Linden 6, 10099 Berlin, Germany; 6 Leibniz Institute for Agricultural Engineering and Bioeconomy (ATB), Max-Eyth-Allee 100, 14469 Potsdam, Germany; 7 Institute of Geomatics, GIS and Remote Sensing (IGGReS), Dedan Kimathi University of Technology, P.O. Box 657-10100, Nyeri, Kenya; 8 Department of Plant and Environmental Sciences, School of Natural Resources, Copperbelt University, Off Jambo Drive, Box 21692, 10101 Kitwe, Zambia; 9 Institute of Environmental Sciences, Hungarian University of Agriculture and Life Sciences, Gödöllő, 2100 Hungary; 10 Universidade Federal de Rondônia, Porto Velho, RO Brazil; 11 Faculty of Applied Computer Sciences, Institute of Geography, University of Augsburg, Alter Postweg 118, 86159 Augsburg, Germany

**Keywords:** Remote sensing, Phenology, DSSAT, Evapotranspiration, CERES-Maize, WaPOR, Climate change, Plant development, Climate sciences, Environmental social sciences

## Abstract

Agricultural production assessments are crucial for formulating strategies for closing yield gaps and enhancing production efficiencies. While in situ crop yield measurements can provide valuable and accurate information, such approaches are costly and lack scalability for large-scale assessments. Therefore, crop modeling and remote sensing (RS) technologies are essential for assessing crop conditions and predicting yields at larger scales. In this study, we combined RS and a crop growth model to assess phenology, evapotranspiration (ET), and yield dynamics at grid and sub-county scales in Kenya. We synthesized RS information from the Food and Agriculture Organization (FAO) Water Productivity Open-access portal (WaPOR) to retrieve sowing date information for driving the model simulations. The findings showed that grid-scale management information and progressive crop growth could be accurately derived, reducing the model output uncertainties. Performance assessment of the modeled phenology yielded satisfactory accuracies at the sub-county scale during two representative seasons. The agreement between the simulated ET and yield was improved with the combined RS-crop model approach relative to the crop model only, demonstrating the value of additional large-scale RS information. The proposed approach supports crop yield estimation in data-scarce environments and provides valuable insights for agricultural resource management enabling countermeasures, especially when shortages are perceived in advance, thus enhancing agricultural production.

## Introduction

The increasing global population and adverse climate impacts are among the main threats to future food security^1^. In sub-Saharan Africa (SSA), the maize output must be increased fourfold to meet its self-sufficiency demand by 2050^2^. Given the future climatic conditions and existing challenges affecting production, the above projected estimate may not be feasible, thus hampering future food security efforts in the continent. Biotic and abiotic influences might result in low production and insufficient food for the rising population in SSA. As a counteractive measure, governments, policy-makers, and researchers should deploy the necessary technologies to better understand and avert the decline in future production while equally maximizing the available opportunities to enhance production^3^. Among these strategies, agricultural production assessment and crop condition monitoring studies at diverse spatial scales are useful for identifying marginally productive areas and identifying opportunities for optimizing production.

Information on crop conditions, growth and production estimates is important to agricultural extension workers, planners, and governments. Such information can be obtained through field experiments and surveys conducted at the household and field levels. Although these approaches have gained increasing relevance in agricultural policy-making, they are costly, especially when conducted at large scales. As an alternative, crop modelling and remote sensing (RS) have been proposed to explore crop conditions and the response of various agronomic measures at relatively low costs^4^. Although these two techniques can be applied independently, their synergistic combination complements their respective strengths, facilitating more robust analysis of agricultural landscapes. For example, RS provides synoptic coverage of the crop status from quantifiable crop condition proxies and estimators of reflectances in specific spectral bands and indices^5^. One important feature of vegetation progression that can be detected through RS is the land surface phenology (LSP). LSP reflects the distinct cyclic pattern of the change in terrestrial vegetation species^6^. Exploring LSP offers opportunities for improving cropland monitoring processes and influences. Similarly, crop models can be used to simulate crop growth and development under diverse environmental conditions and agronomic management practices. The application of these tools focuses more on the biological and physiochemical interactions in the plant‒soil‒atmosphere continuum and entails incorporating several parameters to model various plant processes^7^.

In recent decades, there has been an increase in crop growth simulation models used in agricultural planning and decision-making. The models are categorized widely as carbon-driven, water-driven or radiation-driven based on the driving mechanism^8^. The Decision Support Systems for Agrotechnology Transfer (DSSAT)-CERES-Maize model is one of the simulation models falling under the radiation-driven category as it derives plant biomass from intercepted solar radiation and incorporates the radiation use efficiency (RUE) coefficient^9^. The dynamic simulation model for the maize crop is among the larger family within the DSSAT group of models. The model has shown a remarkable capability for characterizing production in diverse environments^10,11^. It simulates leaf area index (LAI) and root growth variables. It also simulates ET processes, nitrogen dynamics and soil water balance. Furthermore, the model integrates plant-soil-atmosphere processes and outputs variables, including biomass, yield and other variables related to crop production. In simulating the above variables, the model uses algorithms representing various plant, soil and atmosphere processes to describe plant growth and development. The model simulates processes such as ET using the Priestley-Taylor (PT) and FAO-56 Penman–Monteith (PM) options^8^.

Combining crop modeling and RS provides opportunities for assessing crop conditions and production, especially in data-scarce environments. These techniques can be coupled to provide an improved understanding of the underlying environmental processes and cropland status at any given time during the growing season. The advantage of the latter is that it captures the variability in croplands as moderated by climatic effects and socioeconomic factors. In this way, information can easily be integrated into crop modeling frameworks for simulating daily growth and development^12^. Furthermore, Gao et al.^13^ noted the reliability of sensor products for deriving within-season phenological information, including sowing dates.

In existing studies, RS and crop models have been coupled to study maize conditions and development in diverse regions^14–16^. In these studies, various biophysical variables derived from RS have been integrated and assimilated into crop modeling platforms. These assimilation studies have been extensively covered in the literature; however, little research has focused on the retrieval of agronomic management information. Few studies on the derivation of agronomic management information from RS data have confirmed the reliability of data sources in crop model simulations. For example, Rezaei et al.^12^ showed that sowing dates could be derived from Moderate Resolution Imaging Spectroradiometer (MODIS) satellite data, although with minimal uncertainties. Moreover, Leo et al.^17^ showed the significance of RS information in deriving management zone information and optimizing management, for example, fertilizer application using crop modeling. Other researchers have used RS to evaluate management information retrieval^18,19^. However, more research is still needed to improve the estimation of crop model inputs using RS data and to provide a greater understanding of the yield variation at various spatial scales. Furthermore, assessing the model performance in simulating ET at large scales is yet to be addressed. This study, therefore, bridges these research gaps in three aspects. First, the aim of this study is to derive important phenological information from RS and integrate it with the DSSAT-CERES-Maize model for yield prediction. The second objective is to evaluate the integration approach at the sub-county level, the yield-reporting scale in Kenya. The final objective of the study is to evaluate the performance of the DSSAT-CERES-Maize model in simulating ET and examine the implications of the performance on yield prediction at the sub-county scale. The study is important in providing county and national governments and decision-makers in the agricultural sector with an effective approach to yield estimation using state-of-the-art and low-cost technologies towards improved crop production and food security.

## Materials and methods

### Study area

**Figure 1:**
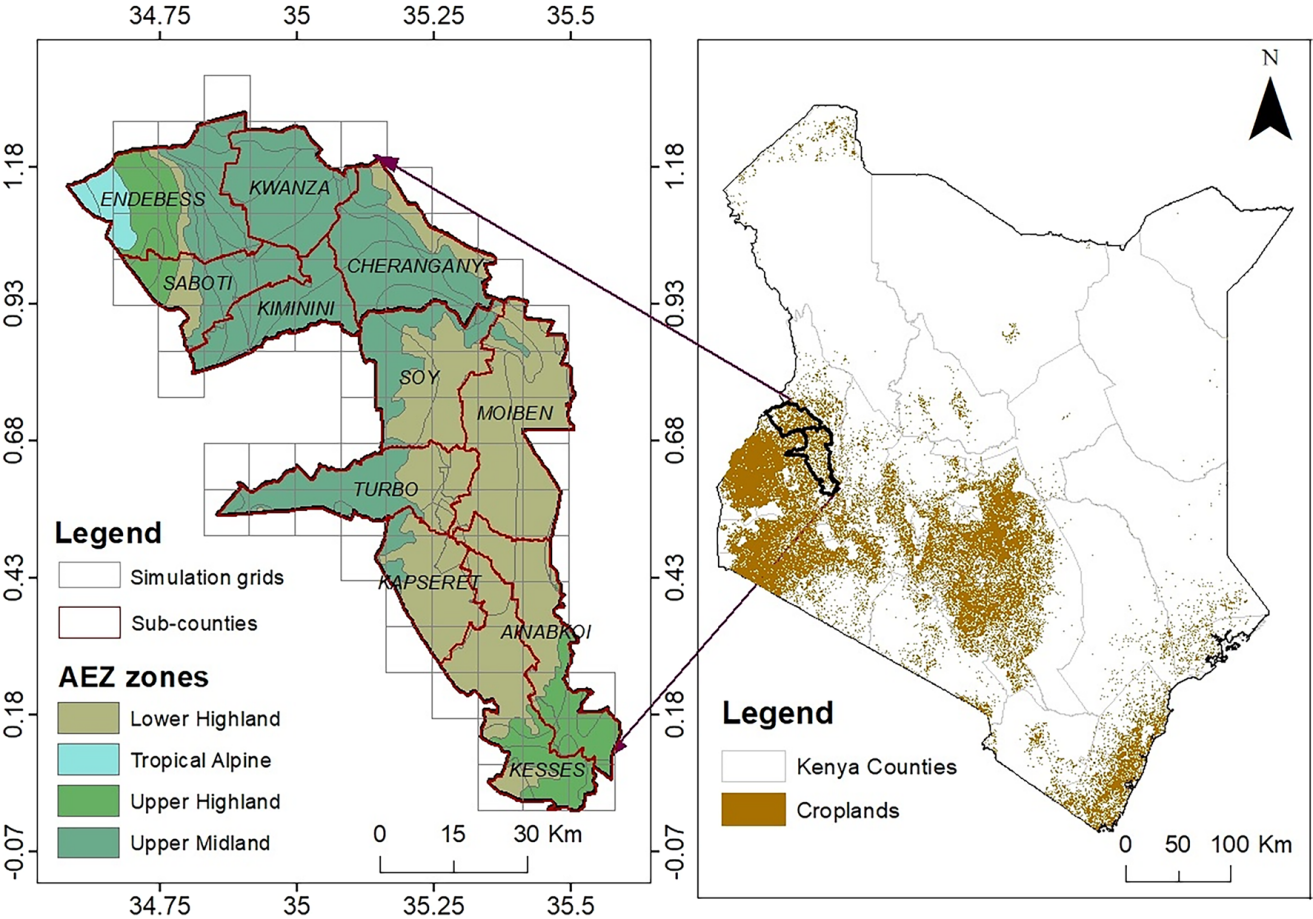
Map of the study sub-counties and simulation grids superimposed on the agroecological zones of the region (left) and the context of the study area in Kenya with an overlay of croplands mask from the Digital Earth Africa (right).

This analysis was performed for the sub-counties of Trans Nzoia and Uasin Gishu Counties in northwestern Kenya (Fig. 1), collectively covering an area of 5430 km^2^ and producing approximately 30% of the total maize harvest in Kenya^20^. Rainfed farming is the dominant cultivation pattern of maize and other crops in the study area. The annual precipitation in the region varies between 900 and 1800 mm. The rainfall pattern is bimodal, with long rains experienced between March and July, whereas short rains occur between October and December. The annual mean temperature varies between 9 and 26 °C. The altitudinal range is high in Trans Nzoia County (1500–4226 m above sea level), while in Uasin Gishu, the altitude ranges from 1500 to 2800 m. The dominant economic activity in the rural and peri-urban areas is agriculture, with crop farming and livestock rearing practised.

### Remote sensing-based land surface phenology

**Figure 2:**
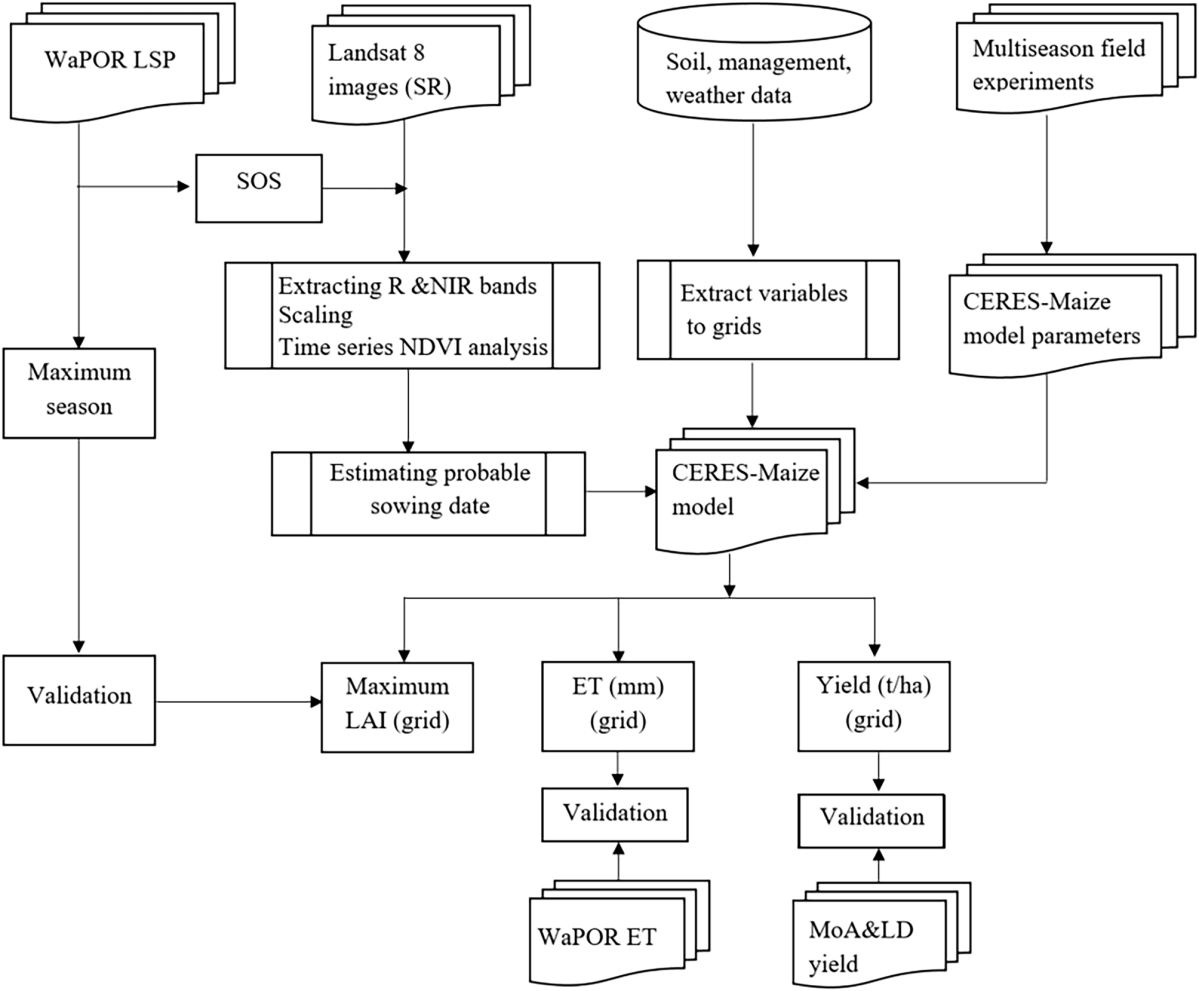
The adopted methodology for the study, where LSP stands for Land surface phenology, ET for evapotranspiration, SR for surface reflectance, SOS for start of the season, WaPOR for Water Productivity Open-access portal, R for Red, NIR for Near Infrared, NDVI for Normalized Difference Vegetation Index, LAI for Leaf Area Index, CERES for Crop Environment Resource Synthesis and MoA&LD for Ministry of Agriculture and Livestock development.

The land surface phenology (LSP) from 2017 to 2021 was derived from RS data. The selected period corresponds with the availability of the observed yield data at the sub-county level to allow for model performance assessment. Satellite LSP derivations can capture the cyclic pattern of the change in terrestrial vegetation^6^. Thus, important indicators of crop growth, such as the emergence date, start of the season (SOS), maximum season (MS), and end of the season (EOS), can be derived. In this study, we utilized the seasonal phenology data obtained from the Food and Agricultural Organization (FAO), delivered through the Water Productivity Open-access portal (WaPOR) portal. The WaPOR portal delivers processed satellite information, including seasonal phenology used to monitor water and land productivity at various regional scales. Multisource LSP data were used to estimate the probable sowing dates at the grid scale. LSP in the WaPOR data is represented by the SOS, MS, and EOS temporal attributes. The data were created from a time series of normalized difference vegetation index (NDVI) composites from Proba-V and Sentinel-2 images. The Proba-V satellite data covered 2009 to 2019. However, from 2019 onwards, the base NDVI layers were derived from the Copernicus Sentinel-2 mission following the decommissioning of the Proba-V satellite. The seasonal cycles of the WaPOR LSP data are delivered using raster files, with each file indicating a dekadal value showing when the respective season was attained. According to the WaPOR data, the NDVI time series covers exactly three calendar years, equivalent to 108 dekads. The target year in the data occurs in the middle (dekad 37–72). Thus, for a given target year, dekad 37 corresponds to the first ten days of January, and dekad 72 corresponds to the last ten days of December. We downloaded and processed the raster files to derive SOS, MS and EOS for each simulation grid. The methodology followed in this study is summarized in Fig. 2.

**Figure 3:**
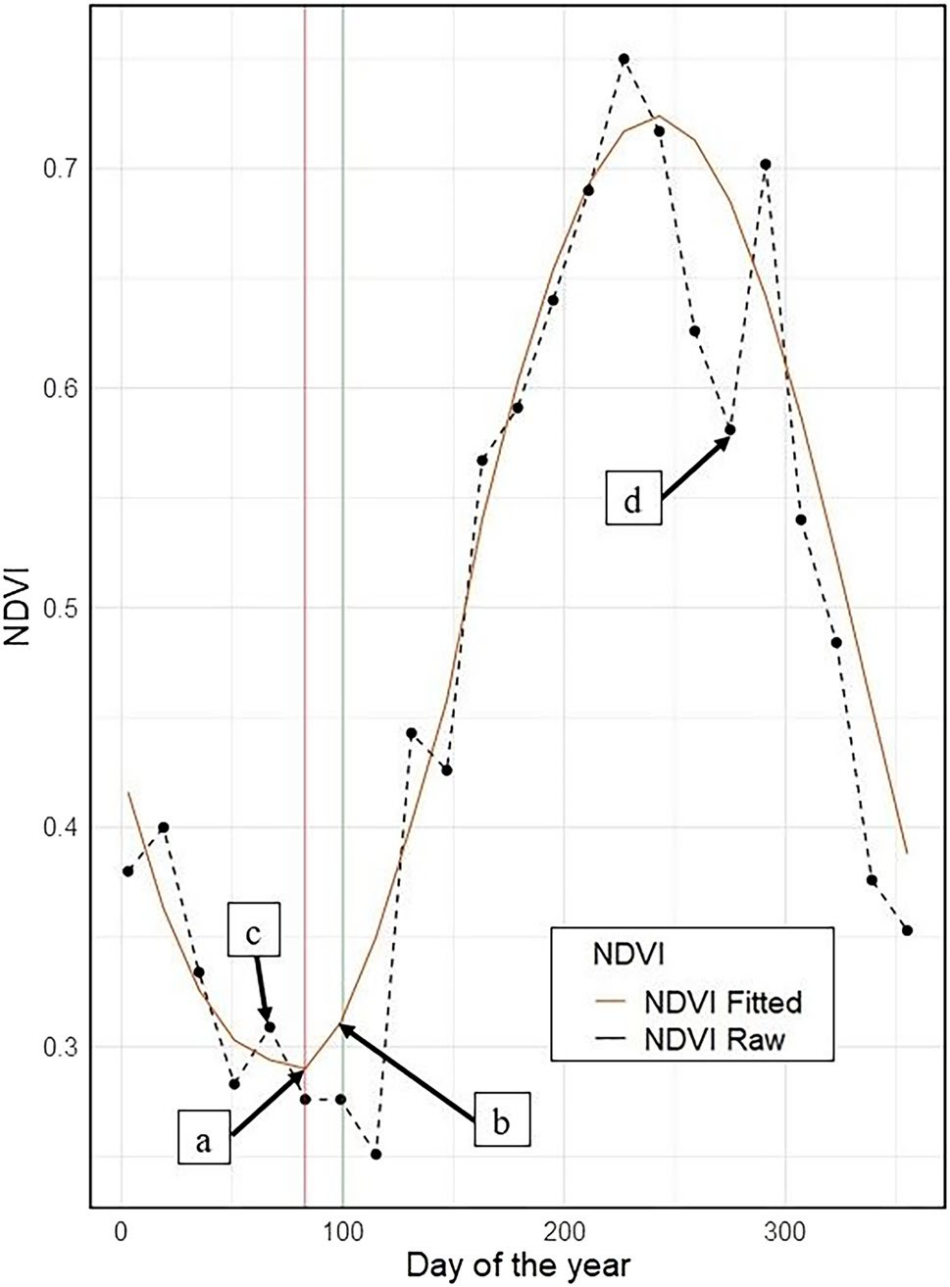
Temporal profile of maize from a selected grid in the study area. The dotted black line shows NDVI raw values and the brown solid line indicates the temporal pattern modeled with the harmonic functions. The vertical red line is the estimated sowing day of the year, and the vertical green line is the median SOS value for the grid derived from the WaPOR data. The label (a) indicates the estimated sowing date, (b) is the median SOS value for the grid, (c) shows a false minor peak before the sowing date, and (d) is a low peak due to low-quality pixels from atmospheric artefacts.

Additionally, time series of the NDVI from the Landsat 8 OLI/TIRS sensor were derived for each grid in the study area. Landsat data provided additional temporal information on crop growth progress before the SOS. This information is crucial to estimate the probable sowing window. We fitted a harmonic function to the time series of NDVI data to overcome the problem of noise and gaps in the time series data. The harmonic function models the temporal signal of the NDVI as the sum of additive and harmonic terms, which are then expressed by phase and amplitude^21^. The analysis of temporal patterns of NDVI was performed in the Google Earth Engine platform^22^. The NDVI and WaPOR datasets were evaluated for the condition that the sowing date in each grid must occur before the SOS, as the SOS indicates the time lag after sowing (Fig. 3). Thus, the sowing date for each grid was estimated as the lowest point on the curve (zero gradient) before SOS attainment. False minor peaks before the estimated probable sowing date were ignored as they could probably indicate the re-growth of weeds after land preparation^23^. As each grid comprises several pixels, the median value was assumed as the probable sowing date for a given grid.

**Figure 4:**
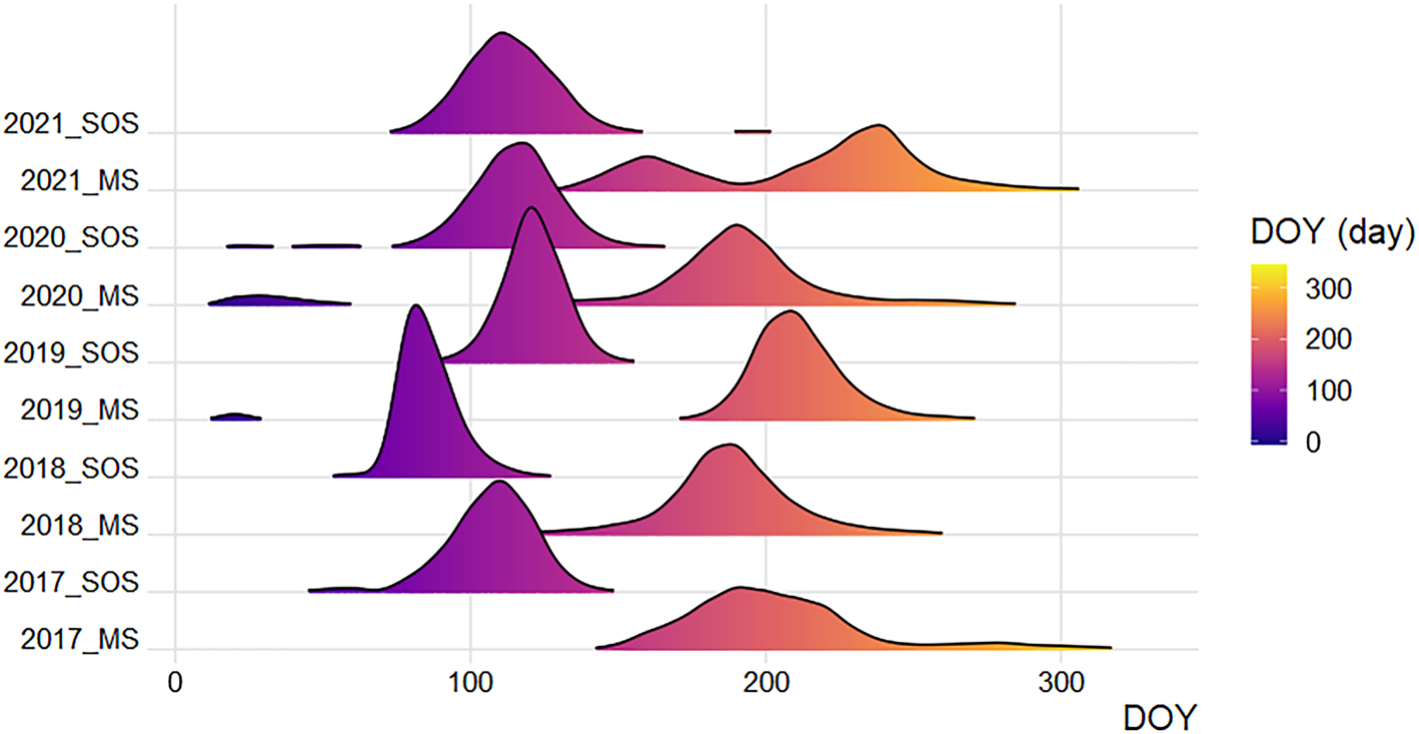
Density distribution for the Start of the Season and Maximum Season values for the 2017–2021 maize growing seasons. The phenological data were extracted for pure maize pixels classified from remote sensing satellite data. The Y axis indicates the SOS and MS in the respective years, and the X axis shows the days of the year (doy). SOS stands for the Start of the Season, and MS stands for Maximum Season.

The derived sowing dates were then used as input to drive the DSSAT-CERES-Maize simulations. The density distribution of the WaPOR SOS and MS for maize pixels in the study area is shown in Fig. 4.

### DSSAT-CERES-Maize modeling

The estimated sowing dates for each grid were used as model input data for the DSSAT-CERES-Maize model. Combined with other model input files, including soil and weather data, the model could be used to simulate the phenology, leaf area index (LAI), biomass and yield during the growing season. For reliable simulations and characterization of crop growth and development, it is paramount that the processes that drive the crop model outputs must be represented as accurately as possible. Thus, the model should be parameterized and evaluated under nonlimited water and nitrogen stress conditions. In a recent study in the study area, on-farm experiments were conducted to parameterize and evaluate the DSSAT-CERES-Maize model^24^. The model was parameterized using observed weather and soil data for 82 fields during two maize-growing seasons in Trans Nzoia County, which is part of the scope of this study. Daily weather variables, including solar radiation, precipitation, and daily maximum and minimum temperatures, were obtained from local weather stations in the study area. Additionally, soil sampling and analysis were conducted in each field to quantify the soil physical and chemical characteristics. The model was run using the parameterized cultivar coefficients to simulate the phenological development, LAI, and yield in the various maize grids. The model simulates phenological development, including the number of days needed to attain anthesis and physiological maturity^25^. These attributes, together with the seasonal progression of the LAI, can be used to infer crop growth progress. In this study, we used the simulated maximum LAI value as an indicator for the peak season. Thus, the value was compared against RS MS observations from archived sources such as WaPOR. Additionally, the simulated yield was aggregated to the sub-county scale and compared to the observed yield to assess the model robustness in representing the yield at larger scales.

### Evaluation of DSSAT-CERES-Maize phenology and yield simulations

The maize phenology simulated by the DSSAT-CERES-Maize model was compared to the MS phenology obtained from RS data during the two seasons. The accuracy metric of the coefficient of determination (R^2^) was used to evaluate the DSSAT-CERES-Maize-simulated and RS-derived LSP values during the peak season. Furthermore, the simulated and observed maize yields were compared at the field and sub-county scales.

### Evaluation of the DSSAT-CERES-model simulated evapotranspiration

In this study, we also assessed the potential of the DSSAT-CERES-Maize model in simulating ET at the grid scale. The soil water balance module of the model is the driver behind ET estimation, usually partitioned into water lost through evaporation and transpiration^26^. The partitioned components are then modified by the extractable water and root growth to determine the crop evapotranspiration (ET). In this study, we adopted the PT method to estimate the potential ET. The technique uses solar radiation and temperature to calculate ET. The method is recommended for humid environments, a characteristic of the study area.

The DSSAT-CERES-Maize ET simulations were compared to the WaPOR retrieved datasets for the study area. The product has been evaluated across Africa with in situ data obtained from multiple eddy covariance (EC) stations and found to exhibit satisfactory agreement^27^. The advantage of this product is that it provides extensive coverage over Africa and the Middle East, enabling low-cost, large-scale assessments in data-scarce environments. Therefore, the cumulative ET over the growing season in each grid was compared to cumulative ET observations from the WaPOR data. Since the WaPOR data are provided on dekadal temporal scales, raster functions were used to compute the WaPOR-derived ET for each grid over the entire season. The evaluation was conducted for all five growing seasons in this study, spanning different weather and plant water availability regimes.

### RS-derived information analysis of the yield and ET simulation results

We assessed the potential of the estimated RS sowing dates and their influence on yield and ET representation across the study area. To achieve this, we generated distributed sowing dates at ten intervals spanning 30 days before and after the estimated sowing date. The window chosen was ideal for capturing both possible early and late sowing dates in the study region. The DSSAT-CERES model was applied to simulate the yield and ET at the six selected dates from 2017 to 2021. The simulated results were aggregated to the sub-county scale by obtaining the mean value in each year. We then used Pearson’s correlation analysis to examine the relationship between the simulated and observed outputs for both variables.

### Ethics declarations

All methods of experimental research and field studies used in this study comply with relevant institutional, national, and international guidelines and legislations. During the field study, our test did not involve endangered or protected species, and no specific permissions were needed for conducting the field study because it was not carried out in a protected area.

## Results

### Evaluation of the DSSAT-CERES-Maize phenology simulations selected seasons

**Figure 5:**
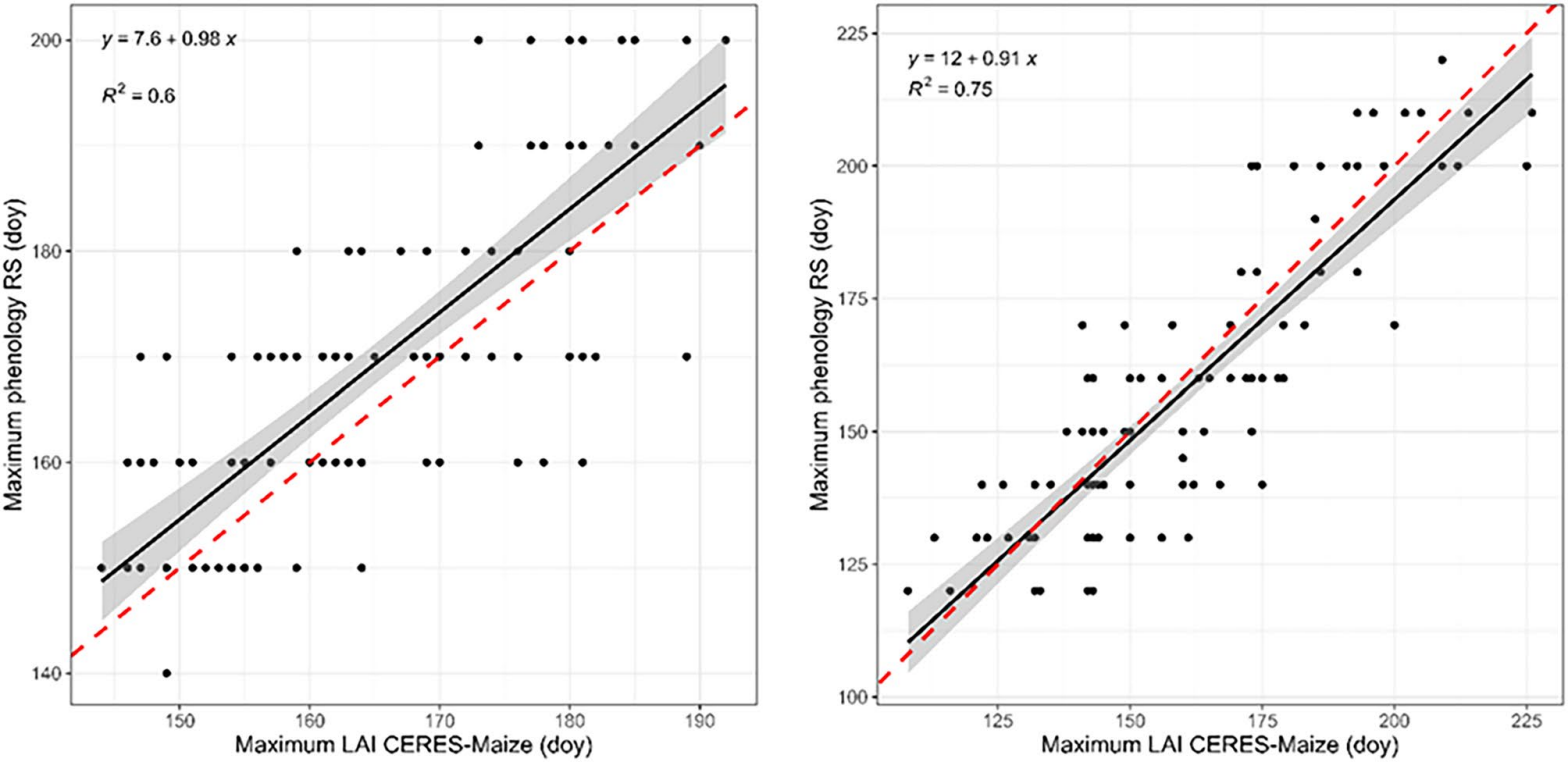
Scatter plots of the correlation between the day of the year for the RS MS LSP and the DSSAT-CERES-Maize-simulated maximum leaf area: (left) 2018 and (right) 2021 growing seasons.

Two maize growing seasons (2018 and 2021) were selected to evaluate the DSSAT-CERES-Maize model-simulated MS values, represented by the maximum LAI. Figure 5 shows the correlation plots between the DSSAT-CERES-Maize modelled MS and WaPOR MS LSP indicator values. The plots reveal a high correlation of the seasonal information between the WaPOR-derived phenology and the DSSAT-CERES-Maize model MS estimations. The correlation coefficient R^2^ between the RS-observed LSP and crop-modelled phenology during both seasons exceeded 0.6, suggesting favourable agreement between the derivation methods. However, the simulated phenology for 2021 exhibited a higher correlation with the RS maximum phenology than that in 2018, which might be explained by the high range of MS values capturing regional phenology dynamics. Nevertheless, the two representative seasons could be used to adequately characterize the MS phenology. Thus, the results of the DSSAT-CERES-Maize model growth phenology were reliable and suitable for informing yield prediction in the region.

### Evaluation of the DSSAT-CERES-Maize yield simulations

**Figure 6:**
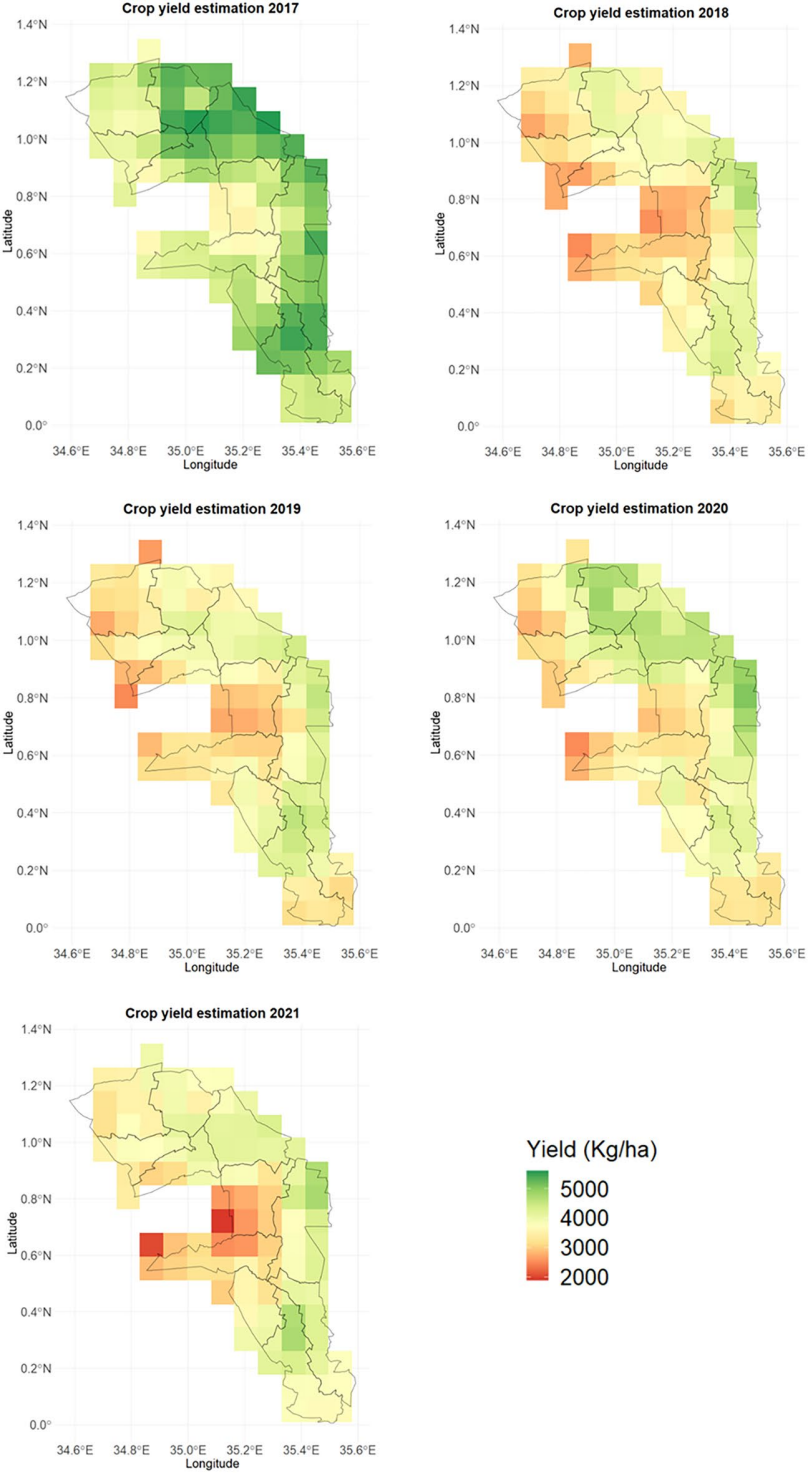
Simulated maize yield across various sub-counties for the 2017–2021 period.

**Figure 7:**
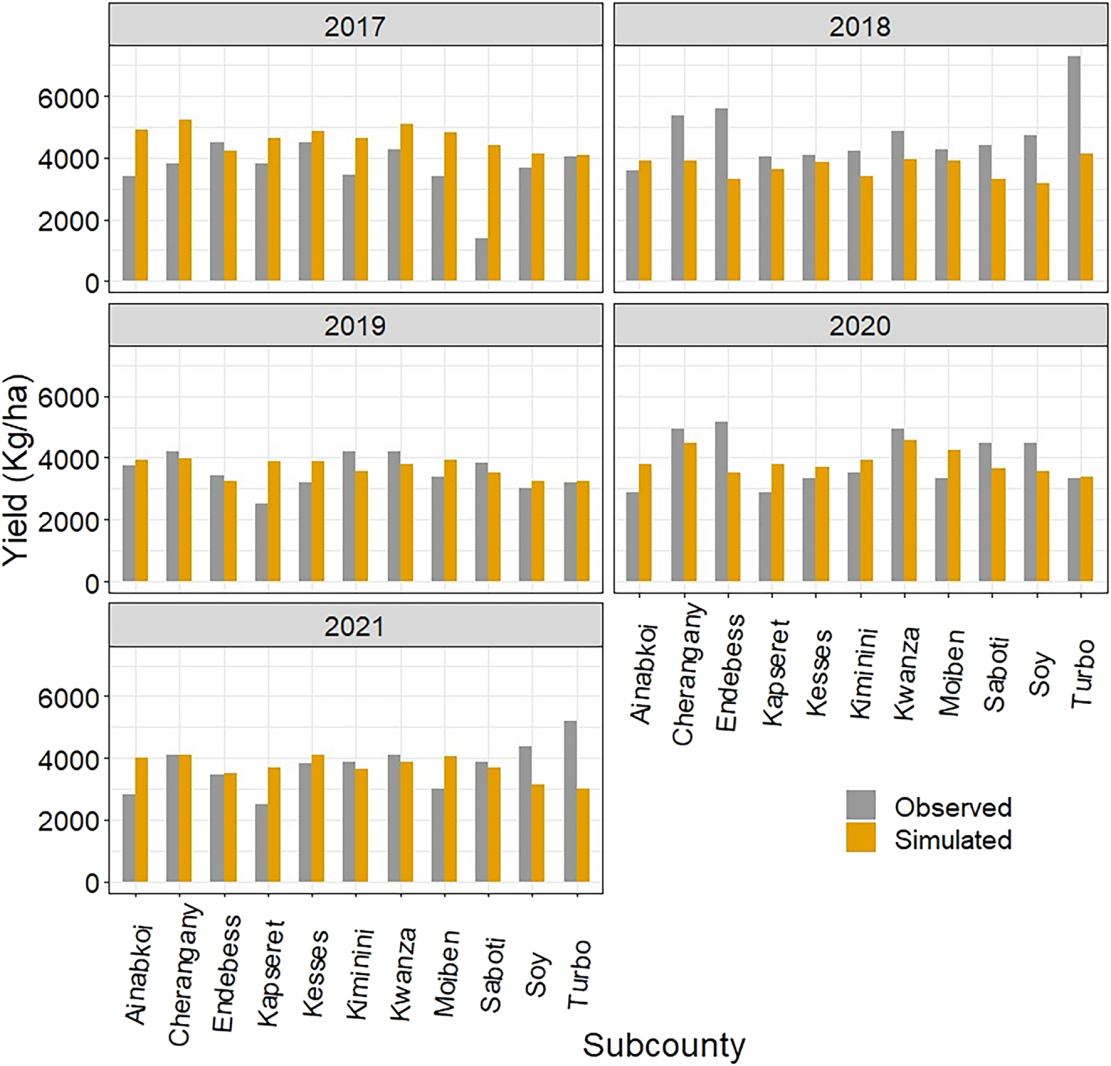
Comparison of the observed average yield with the simulated average yield in the various sub-counties for the 2017–2021 period.

The DSSAT-CERES-Maize gridded simulations (Fig. 6) were aggregated to the sub-county level and compared with the reported yield records from the Ministry of Agriculture and Livestock Development (MoA&LD)^20^. The results showed that the DSSAT-CERES-Maize model suitably reflected the reported harvests in the various sub-counties during the different growing seasons (Fig. 7). The agreement was satisfactory in 2017 and 2019 in most sub-counties. There was a slight difference between the simulated and observed yields in 2018 and 2020. In 2021, maize production was characterized well in the humid sub-counties of Trans Nzoia County, whereas the sub-counties in Uasin Gishu revealed low to moderate yield differences.

The yield overestimations and underestimations of the DSSAT-CERES-Maize model were notable. In 2017, the model slightly overestimated the yield in most counties (Fig. 7). The yield was underestimated in almost all sub-counties in 2018. Despite the slight underestimation of the yield, the production was suitably characterized in 2019, especially in the humid sub-counties of Trans Nzoia County. However, the yield in Uasin Gishu County was overestimated in most sub-counties. A similar pattern was observed in 2020, although a slight difference between the modeled and observed yields was observed. The yield was characterized well in 2021, except for a few sub-counties in Uasin Gishu County. The DSSAT-CERES-Maize model demonstrated reliability in simulating yields across most counties and growing seasons.

### Evaluation of DSSAT-CERES-model evapotranspiration simulations

**Figure 8:**
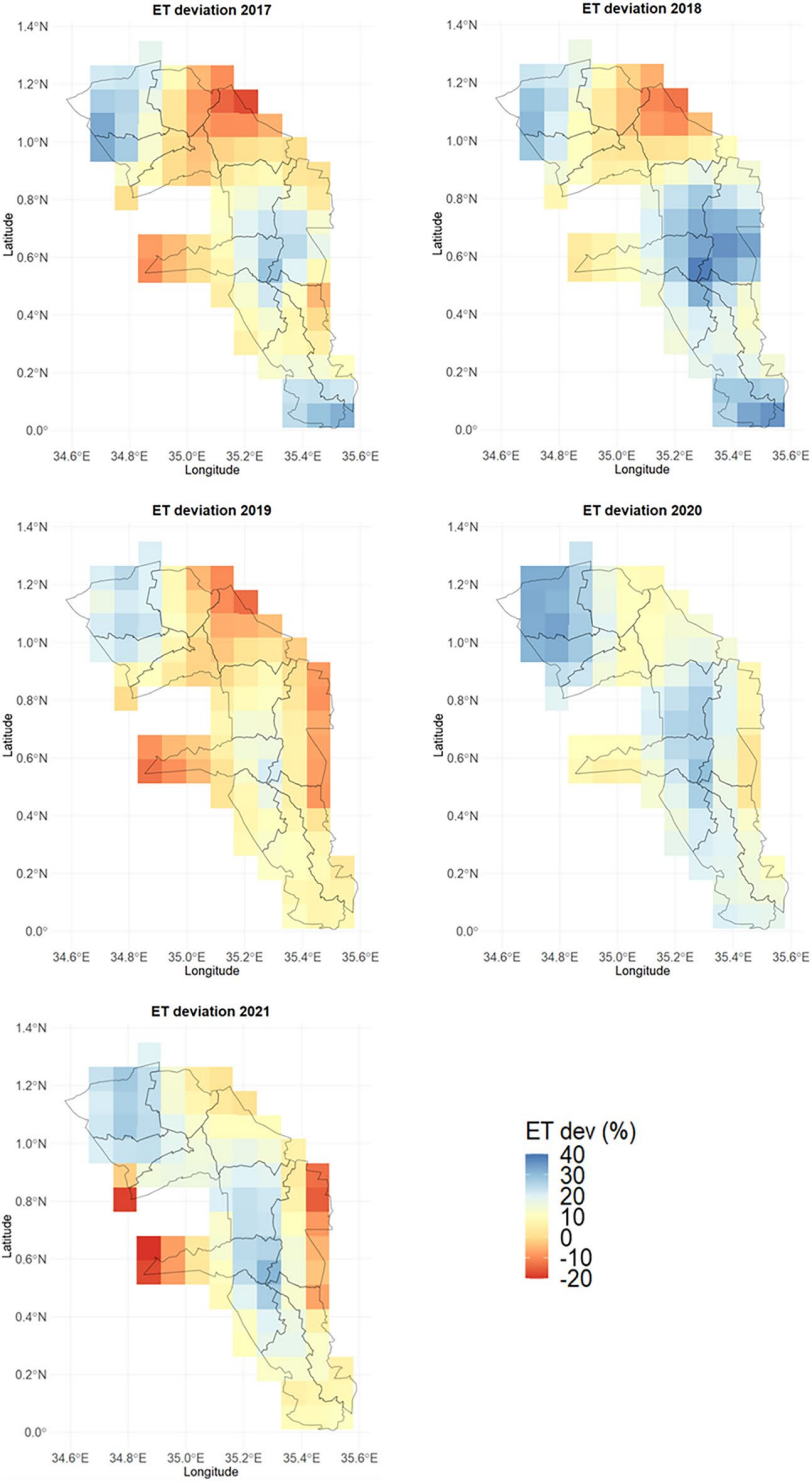
ET deviation (%) between the simulated DSSAT-CERES-Maize ET and WaPOR ET values.

The WaPOR ET data were compared with the DSSAT simulated ET values to establish the model reliability in characterizing ET across space and time. Subsequently, the deviation between the model simulations and the WaPOR data was mapped for every simulation grid. The results showed minimal to medium deviations between the sources across the study region, which also varied during the various growing seasons (Fig. 8). Slight deviations (± 10%) were notable in 2017 and 2019. In contrast, the deviation between the WaPOR and the model-simulated ET values was high in 2018 and 2020. In Soy and Turbo sub-counties in Uasin Gishu, high deviations of > 20% were recorded. Additionally, in the sub-counties in Trans Nzoia County, for instance, Endebess and Kwanza, slightly high deviations were obtained. The findings on the basis of ET evaluation are closely related to the performance of yield prediction. For example, the high yield simulation performance in 2017 and 2019 is associated with slight deviations of the ET estimates. In contrast, the poor yield simulations in 2018, 2020, and 2021 could be attributed to poor ET estimation by the crop model, as shown by the large deviation from the WaPOR data. The evaluation of the simulated ET values suggests that accurate estimation of ET and plant‒soil water exchange is crucial for yield prediction.

### RS-derived information analysis of the yield and ET simulation results

**Figure 9:**
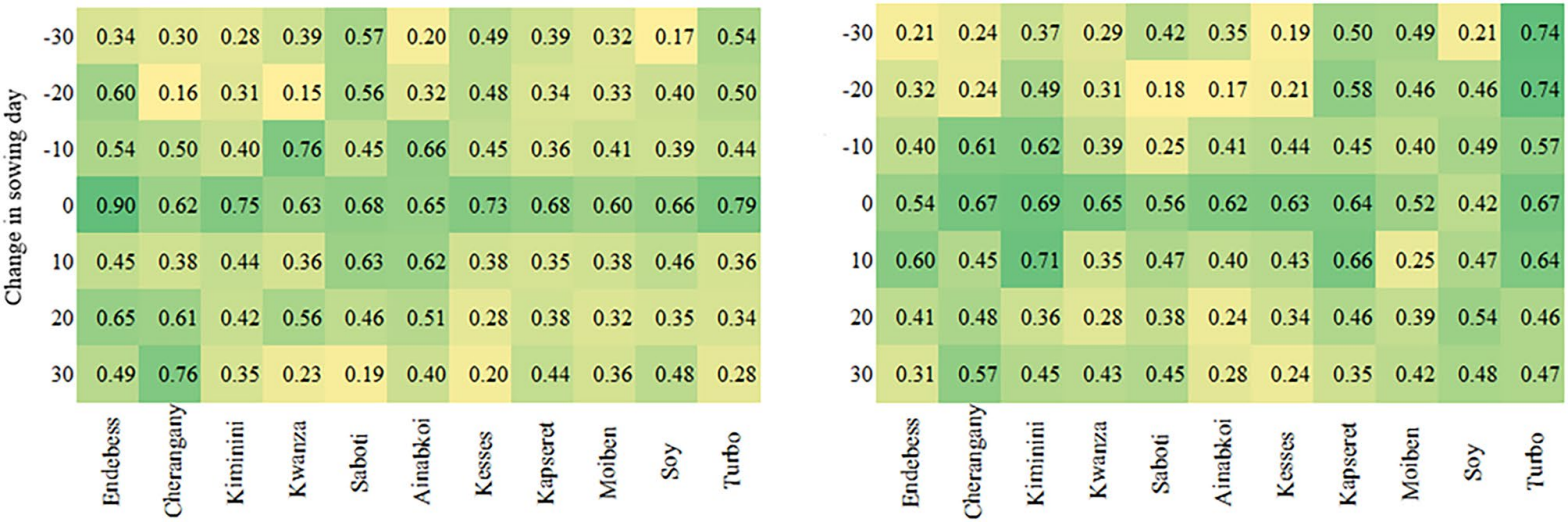
Correlation coefficients between (left) the simulated and observed yields and (right) simulated and WaPOR ET values based on the RS-estimated sowing date (baseline) and ± 30 days from the estimated date across the various sub-counties.

The correlation plots (Fig. 9) show that the yield and ET predictions are satisfactory when sowing information estimated from remote sensing data is applied. This is indicated by the high correlation between the observed and simulated variables. The performance of the DSSAT-CERES-Maize model in characterizing the yield under the provision of RS-estimated dates was favourable (the correlation coefficients varied between 0.6 and 0.9) across the sub-counties. Similarly, the ET simulation performance under the influence of RS-estimated dates varied between 0.42 and 0.68. There were notable differences in the yield and ET simulations for the other modeled dates. A low correlation between the observed and simulated variables was particularly evident with increasing departure from the RS-estimated date. The results further showed better correlations in regions with humid conditions. The best correlation coefficient (r value = 0.9) for the yield occurred in the Endebess sub-county, which is relatively humid.

Regarding ET, the highest correlation was achieved in the Turbo sub-county with moderate humid conditions. Despite the good relationship between the observed and the simulated ET and yield, especially for the RS-derived sowing dates, the correlations were low. This is because the correlation analysis was conducted based on the aggregated yield at the sub-county scale and different years. In this instance, the data points used for correlation were fewer, and small differences in the yield observations and simulations can yield poor comparisons. Nonetheless, proper estimation of crop models driving variables can be used to predict yield and ET accurately at sub-county scales. Furthermore, the results show that driving model simulations with poorly estimated information leads to compromised prediction accuracies.

## Discussion

### Phenology characterization and integration in the crop modeling framework

Our study provides a yield prediction approach integrating RS information and crop modeling to derive agronomic management information and to further improve maize yield estimation in Kenya. The study assessed the robustness of estimating sowing dates based on SOS and MS phenology information derived from RS data. This is founded on a strong link between RS-derived parameters and seasonal phenological cycles obtained from locally calibrated process-based models^28^. In addition, we assessed the reliability of the derived management information approach by evaluating the simulated yield at the sub-county level. The above information is critical to inform food production management and security planning at downscaled spatial scales, for example, grid scales.

Our findings indicate suitable agreement between the RS-derived phenology and simulated maize growth results. This is indicated by the satisfactory accuracy evaluation metrics during the maize peak season derived from both techniques. The accuracy of peak season derivation from crop modeling frameworks highly depends on the accuracy of the sowing dates^29^. Other researchers similarly noted the application of RS-derived SOS as crucial in estimating sowing dates for obtaining crop model inputs^30,31^. The variation in sowing dates across agricultural landscapes leads to distinct phenological characterization, influencing the yield and other outputs retrieved from modeling platforms. Furthermore, varied sowing windows influence plant–weather interactions, especially in rainfed cropping systems^32^. Therefore, accurate estimation of this information is useful for determining the prevailing seasonal crop conditions and progression in agricultural landscapes.

### Evaluation of the DSSAT-CERES-Maize yield simulations

Yield simulation involving more reliable agronomic management estimations from WaPOR data is integral for multiscale analysis. Our results indicated suitable maize yield simulation at the sub-county scale resulting from the improved approximation of sowing dates. The findings corroborate those of previous studies, including^33–35^, who obtained suitable yield estimates at the regional and national scales using integration approaches of crop modelling and RS phenology. Evaluation of the simulated yield at finer scales, for example, at the grid scale, was impossible due to the lack of observed data in Kenya. Nonetheless, the presented findings are crucial for evaluating the integrated approach in areas with sufficient gridded observations. The maize yield was characterized well across the years, and the maize performance differed among the studied sub-counties. The performance was attributed to the high variability in soil and weather conditions across the sub-counties, also characterized by different agro-ecologies. The results showed that the DSSAT-CERES-Maize model could capture diverse conditions across the growing seasons. The accuracy of the model in representing maize conditions across similar spatial scales has also been indicated in other studies in Kenya and other SSA production systems^36,37^. Despite the attained overestimations in a few sub-counties, the model simulated the maize yield accurately, and the simulations indicated a better year in terms of production. The overestimation of yield could also result from the failure of the model to account for yield reductions due to biotic factors, for instance, the fall armyworm invasion, a common phenomenon in the region^38^. The conditions in each year were substantiated by food security outlook reports from the Famine Early Warning Systems Network^39^, indicating reliable off-season rains in July in the study area. Usually, this month coincides with the MS in the region, and the sufficient soil moisture at the vegetative tasselling and silking stage causes improvement in flower formation and pollen fertilization and an increase in the grain-filling duration, leading to high yields.

Furthermore, the model could be used to simulate high yields in most sub-counties in 2020, conforming with the respective observed yields. The year was characterized by enhanced rainfall between March and May during the long rainy season^40^. The model, however, did not capture the yield well in some humid sub-counties, which is also reflected by the high ET estimation deviation between the model and WaPOR data. Accurate water balance determination allows satisfactory crop development and growth prediction, especially under humid conditions^41^.

### Evaluation of the DSSAT-CERES-model evapotranspiration simulations

The ET model simulations revealed low to medium deviations from the WaPOR ET data in most of the study area. This demonstrated that the DSSAT-CERES-Maize model is suitable for examining water and energy fluxes in maize cropping systems. The deviation in the northeastern sub-counties of the study area was lower than that in the humid western and southern zones. In addition to 2020, the deviation between the model-simulated and WaPOR ET values was low. The year 2019 revealed low ET deviation in most sub-counties, matching the high agreement between the simulated and observed yields. Certain sub-counties, for example, Kwanza and Cherangany, exhibited a relatively low deviation and were also among the regions whose yield was well represented across the years. Evapotranspiration was poorly represented in humid zones (western and southern regions) and in part of the central region of the study area. The western region lies in the high-altitude zones of the Mt. Elgon ecosystem, which experiences high humidity and low evapotranspiration rates^42^. The ET deviation in this zone was high, and the DSSAT-CERES-Maize model could not capture the ET dynamics well in the region. This is also confirmed by the underestimation of the yield by the model in four out of the five seasons studied. A similar finding was also obtained by^43^, who observed low yield and biomass simulation results under humid conditions.

Similarly, some parts of the Moiben and Soy sub-counties with relatively low rainfall showed high deviation in the ET estimation results. The area is also dominated by Ferralsols, with high permeability and poor chemical composition, which might be the reasons for the high deviation in the ET values^44^. The model simulations and the WaPOR ET data agreed well, as reflected in the predicted yield across the various sub-counties and growing seasons.

### RS-derived information analysis of the yield and ET simulation results

The study shows that RS-derived variables are reliable for parameterizing crop models at the grid level and for predicting the yield at the sub-county scale, as indicated by the favourable agreement between the DSSAT-CERES-Maize and WaPOR-derived phenology data. The RS-derived phenology coupled with additional Earth observation data enabled the estimation of sowing information at the grid level. Rezaei et al.^12^ found that RS information can facilitate an improvement in the estimation of sowing dates for crop modeling purposes. Additionally, Sadeh et al.^45^ applied RS data to detect sowing dates across Australia and reported reliable model outputs. Moreover, Urban et al.^18^ used multisource RS data to extract the phenology and sowing dates in the United States (US) and found that satellite techniques were reliable in capturing field information. Diao^46^ characterized the phenology of corn and soybean and achieved suitable accuracy in obtaining the transitional dates of different years. RS-derived information is indispensable in enriching crop models and improving their outputs in data-scarce environments^47^. Specifically, phenological information can lead to improved intracropland thematic detail distinction and better estimation of yields and crop area statistics^48^. Yield estimations can be further improved by coupling the DSSAT-CERES-Maize model with other RS-based information, such as leaf area index and biomass^49,50^.

The present study reveals a satisfactory performance of the DSSAT-CERES-Maize model in simulating the yield and ET at the sub-county scale with the integration of large-scale RS information. The model performance was evaluated at the sub-county scale. This was limited by the availability of yield data given this spatial coverage provided by the ministry responsible for crop production in Kenya^51^. Nonetheless, our results demonstrate the potential for reducing uncertainties associated with process-based crop models by improving poor-quality and coarse data inputs associated with crop models at the grid scale. The study results could contribute to revealing yield gap areas and suggesting management options for optimizing production.

## Conclusion

The study provides valuable insights into how phenology can be used to derive crop model input information for improving yield prediction at sub-county scales. The study concludes that remote sensing (RS) information is crucial for determining agronomic information in the study area. In particular, the information can be used to obtain distributed sowing practices, which can serve as crop model inputs for yield prediction and effective decision-making for enhancing production. Furthermore, the study results indicated the reliability of the DSSAT-CERES-Maize model in simulating evapotranspiration (ET) across the region and during various growing seasons. Areas with better ET characterization also exhibited accurate yield predictions. To enhance the assessment of seasonal crop conditions and crop yields, agricultural extension officers and departments at the sub-county and county levels should integrate user-friendly and freely available crop modeling platforms such as the DSSAT-CERES-Maize model and readily available RS information, such as the WaPOR products. These approaches could provide cost-effective ways to evaluate production across various scales. Implementing these methods significantly contributes to a better understanding of crop conditions, growth and their driving factors, which is crucial for addressing yield gaps and adapting production strategies to changing climatic conditions. We recommend extending this approach to other regions to facilitate the formulation of effective policies and strategies for enhancing agricultural production.

## Data Availability

The DSSAT-CERES-Maize model parameters used in this study are accessible at 10.1007/s42106-022-00220-5. Other datasets are freely available from the respective data portals. Remote sensing phenology data can be accessed from the FAO portal to monitor water productivity through open access of remotely sensed derived data (WaPOR) https://wapor.apps.fao.org/catalog/WAPOR_2/2. The global high-resolution soil profile dataset can be obtained from the Harvard University dataverse website https://dataverse.harvard.edu/dataset.xhtml?persistentId=doi:10.7910/DVN/1PEEY0. The precipitation data can be accessed from the CHIRPS website (ftp://ftp.chg.ucsb.edu/pub/org/chg/products/CHIRPS-2.0/). The Daily solar radiation was obtained from the National Aeronautics and Space Administration website https://power.larc.nasa.gov/. The daily minimum and maximum temperatures were synthesized from the global dataset of the daily maximum and minimum near-surface air temperatures over land accessible from 10.25380/iastate.19714901.v2 for 2017–2020 simulations and the NASA POWER website for 2021 simulations.
